# Chaotic itinerancy within the coupled dynamics between a physical body and neural oscillator networks

**DOI:** 10.1371/journal.pone.0182518

**Published:** 2017-08-10

**Authors:** Jihoon Park, Hiroki Mori, Yuji Okuyama, Minoru Asada

**Affiliations:** 1 Department of Adaptive Machine Systems, Graduate School of Engineering, Osaka University, Suita, Osaka, Japan; 2 Department of Intermedia Art and Science, School of Fundamental Science and Engineering, Waseda University, Tokyo, Japan; 3 Systems Intelligence Division, Open and Transdisciplinary Research Initiatives, Osaka University, Suita, Osaka, Japan; Tianjin University, CHINA

## Abstract

Chaotic itinerancy is a phenomenon in which the state of a nonlinear dynamical system spontaneously explores and attracts certain states in a state space. From this perspective, the diverse behavior of animals and its spontaneous transitions lead to a complex coupled dynamical system, including a physical body and a brain. Herein, a series of simulations using different types of non-linear oscillator networks (i.e., regular, small-world, scale-free, random) with a musculoskeletal model (i.e., a snake-like robot) as a physical body are conducted to understand how the chaotic itinerancy of bodily behavior emerges from the coupled dynamics between the body and the brain. A behavior analysis (behavior clustering) and network analysis for the classified behavior are then applied. The former consists of feature vector extraction from the motions and classification of the movement patterns that emerged from the coupled dynamics. The network structures behind the classified movement patterns are revealed by estimating the “information networks” different from the given non-linear oscillator networks based on the transfer entropy which finds the information flow among neurons. The experimental results show that: (1) the number of movement patterns and their duration depend on the sensor ratio to control the balance of strength between the body and the brain dynamics and on the type of the given non-linear oscillator networks; and (2) two kinds of information networks are found behind two kinds movement patterns with different durations by utilizing the complex network measures, clustering coefficient and the shortest path length with a negative and a positive relationship with the duration periods of movement patterns. The current results seem promising for a future extension of the method to a more complicated body and environment. Several requirements are also discussed.

## Introduction

Nonlinear dynamical systems often show a phenomenon, called chaotic itinerancy, in a state space, where the state of the system transits along a certain trajectory consisting of stable (attractors) and unstable ones. From this perspective, the diverse behavior of animals and its spontaneous transitions could be explained by a complex coupled dynamical system consisting of a physical body and a brain. The main question is what a key element is and how it works for the emergence of the diverse behavior. In this study, we attempt to obtain a new insight into the potential of the coupled dynamics between a physical body (i.e., a snake-like robot) and a brain (i.e., a nonlinear oscillator network) based on the behavior and network analyses with measures for complex networks.

The spatiotemporal complex interactions of neurons or regions in the brain lead to different networks from the one given at the beginning, which are estimated based on the statistical analysis of correlation or causality between brain signals, differing from the anatomical network. The brain network herein refers to two kinds of networks: wired network based on the physical connectivity of neurons, and information network based on the statistical analysis of the connectivity between neurons during spatiotemporal complex interactions. Imaging studies using functional magnetic resonance imaging (fMRI), electroencephalography (EEG), and magnetoencephalography (MEG) measure the activities of the human brain depending on experimental tasks [1–4], and information networks could be constructed based on the statistical measure, such as correlation or causality between brain regions.

Biological [5, 6] and computational [7, 8] studies have shown that information networks are closely related to wired networks. The importance of wired and information networks for cognitive function or variability of behaviors has been discussed in studies on autism spectrum disorder [9] and the motor development of infants [10]. However, an issue about the relationship between the information network estimated from the statistical measure for brain activities and the behavior that emerges from the coupled dynamics between the physical body and the brain has been left unsolved. With regard to this issue, we focus on a network to understand the emergence of the behavior from the perspective of diversity, and the information structure behind each movement pattern and their transitions from the viewpoint of the sustainability of movement.

The computational modeling of brain activities has been studied at various levels ranging from intra- [11, 12]and inter-cell [13] interactions to brain regions [14, 15]. Izhikevich and Edelman [16] developed a large-scale computational model of mammalian thalamocortical systems with biologically detailed structures to understand the complex dynamics caused by a neuronal process in the brain. Based on this model, they showed that various spontaneous activities emerge through synaptic plasticity and interaction between neurons. Abstracted models that represent the brain region as an oscillator with fewer parameters than a detailed model have been also been studied [15, 17]. Cumin and Unsworth [18] proposed an abstract model to explain the synchronizations in the brain using the Kuramoto model [19, 20]. They showed that the synchronization resulting from the interaction between oscillators is affected by connectivities and the coupling strength between the oscillators. However, understanding neural mechanisms, from which diverse bodily behavior emerges, from the simulation of neural activities alone without a physical body is difficult.

A dynamical system approach was used to explain the complex interaction process between the brain and the body in an environment in artificial intelligence [21], cognitive science [22–24], and developmental psychology [25–27]. Kelso [28] used the metastability concept to explain the emergence of behavior patterns and their transitions. From this viewpoint, behavior patterns are self-organized from the interaction between the brain and the body in an environment. Their states and transitions are represented by attractors and trajectories in a state space. Chaotic itinerancy, which is a similar concept, but is more theoretical, especially with respect to the instability of attractors, has been proposed to represent the dynamics of the brain. Chaotic itinerancy refers to the transitions among multiple attractors in a high-dimensional state space [29]. The human brain and the sensory organs of animals may exhibit chaotic itinerancy [30, 31]. However, how chaotic itinerancy can emerge from the interaction between a brain and a body is unclear.

Inspired by the concept of chaotic itinerancy, Kuniyoshi and Suzuki [32] proposed a computational model consisting of musculoskeletal and neural systems. This model facilitates the emergence of different movement patterns through an interaction with an environment. In their model, adaptive behaviors emerge immediately through the mathematical model of the coupled map lattice (CML) and the globally coupled map (GCM) of chaotic elements. Each CML receives feedback signals from the muscles of the body and sends output signals to these muscles. These CMLs globally interact through a GCM. Furthermore, they showed different movement patterns, including a goal-directed behavior that emerged through body constraints, such as the alignment of muscles or an object attached to the body. A detailed infant [33] and fetus model [34, 35] was proposed as an extension study of [32]. These studies showed that the interaction between the brain and the body in an environment can generate infant- and fetus-like whole-body movements. However, these studies did not focus on the coupled dynamics behind emergent behaviors and their transitions.

In this study, we adopt such a dynamical system approach to understand neural and behavioral dynamics from the embodiment system for the emergence of a diverse behavior and its transitions. From the perspective of existing studies that the connectivity of wired networks may be related to behavioral variability [10, 36], we focus on understanding the relationships between the emergence of a diverse behavior and nonlinear oscillator networks and that between behaviors and an information network. The remainder of the paper is organized as follows: we provide a more detailed background on behaviors and the neural dynamics of the brain in the following section. Next, we introduce the snake-like model, non-linear oscillator networks, and experimental settings. We then describe the analytical methods for the behavior and the information network, and show the experimental results. The following section provides a more detailed background and methods for the behavior and neural network analyses. Finally, the discussions and future scope are given.

## Behaviors and neural dynamics of the brain

### Wired and information network structures in the brain

Brain-imaging techniques with complex network theory have been applied to analyze the relationship between the structure of a wired network (anatomical network) and the dynamics of neural activations. Sporns et al. [37, 38] and He et al. [39] applied the complex network theory to the analysis of observed data in imaging studies to comprehend the structure of the wired network of the brain. Recent DTI analyses using the complex network theory revealed that the brain has a complex network [40]. The connections among the nodes of this complex network are neither purely regular nor purely random, and the degree of connection has a heavy-tailed distribution. Small-world [41] and scale-free networks [42] are typical complex network structures. The complex network structures are observed in real-world systems, such as the World Wide Web, social networks, and the brain.

The brain exhibits various dynamic activities from the spatiotemporally complex interaction of neurons and regions in the brain under different situations. Buzsaki and Draguhn [43] and Buzsaki and Watson [44] showed that the brain exhibits rhythmic neural activities (oscillations) with different frequencies and scales, which cause various rhythmic patterns, such as synchronized and desynchronized patterns among oscillators. The relation between these oscillators estimated by using correlation or causality leads to a virtual structure different from a wired network, which is called functional networks [1–4], but here we call it information network as explained in the Introduction. Information networks from the relation of activities in the brain using signals from EEG, MEG, or fMRI are dynamically changing with a fast time scale, as compared to anatomical connectivity and it varies depending on the given task or current circumstances of agents. The structure of the information network may possibly express the change of the coupled dynamics, including the sensory motor system and nervous systems. Studies using fMRI [45–47] and MEG [48] showed that the information network has complex network properties. However, how the wired network and the body are related in the emergence and transition of behavior, and the characteristic and role of the information network in this are still not clear.

### Diverse behavior and network in the brain

For living things to adapt to various environments, it is important to choose appropriate behaviors from the diverse repertoire of behaviors. Moreover, from the viewpoint of the motor developmental aspect, the diversity of spontaneous movement patterns observed in early childhood affects subsequent motor development. Hadders-Algra discuss that an atypical motor developmental child has a problem of restricted movement variability or limited variation. They argued it may result from a difference in the cerebral connectivity [10].

Many studies have addressed the importance of wired and information networks to understand how cognitive functions and motor behaviors develop [9, 49–53]. Connectivities in networks are changing with brain development, and a changed connectivity would induce a different relationship of dynamics between the brain and the body. Hadders-Algra also discussed the relationship between the emergence of a varied and complex movement of fetus and cortical subplate which contributes to build the thalamic-cortical path way [54]. This might imply the change of the coupled dynamics by forming the interaction of the dynamics of body and the nervous system, but the understanding of how bodily chaotic itinerancy emerges from the dynamics of the nervous system and the body with different constraints and time scale is not yet sufficient. We examine herein the behavioral difference from the wired network structure and the change of the interaction between the body dynamics and the nervous system by using the structure of the nonlinear oscillator network and the degree of coupling between body and network as variables.

### Chaotic itinerancy from coupled chaotic elements and phenomenon in the brain

Chaotic itinerancy is a trajectory of successive chaotic transitions from a quasi-attractor to another one in a high-dimensional state space [29]. This phenomenon is observed in coupled map lattice (CML) and global coupled map (GCM) models, which are proposed to constitute complex nonlinear systems [55]. The transitions between ordered and disordered patterns in these models emerge in an intermediate region between the fully regular and chaotic regions. Similar phenomena were also reported in [56, 57].

The human brain and the sensory organs may exhibit the dynamics of chaotic itinerancy. For example, Freeman observed such state transitions in animal olfactory systems [30] and in human EEG studies conducted during sleep [31]. Based on these phenomena, Tsuda et al. proposed a computational model to describe chaotic itinerancy in the brain [58–60], which shows that this transitory dynamics can be regarded as a chaotic switch between the synchronized and desynchronized states of neurons. The model is also used to explain the relationship between chaotic itinerancy and cognitive functions at the conceptual level [61, 62]. The model proposed in these studies is expected to be a theoretical infrastructure for the main issue of dynamic interaction in this article. We examine herein how bodily chaotic itinerancy can emerge from the interaction between the body dynamics from the mechanical interaction of multiple links with muscles and the neural dynamics from the electrical signal interaction of spontaneous activities of neurons.

### Emergence of adaptive behaviors as chaotic itinerancy

As mentioned above, inspired by the concept of chaotic itinerancy, Kuniyoshi and Suzuki [32] showed the emergence of adaptive behaviors from the coupled chaotic elements through the body in an environment. Based on this model, Kuniyoshi and Sangawa [33] and Mori and Kuniyoshi [34] extended the structure of the physical body to a more complicated model, that is, the body of a fetus consisting of 198 muscles with tactile sensors. They showed that the musculoskeletal and neural systems interact with each other in the uterine environment and. As a result, certain types of ordered movements and their transitions were observed. The abovementioned studies ([32–34]) showed the importance of embodiment with regard to the spontaneous emergence of both behaviors and their transitions.

Yamada et al. [35] recently constructed a detailed brain-body-environment system of a fetus to understand cortical learning via sensorimotor experience in a uterine environment. In this model, more detailed anatomical and physiological data were used for a musculoskeletal system, tactile sensor, vision sensors, a uterine environment, and a cortical model. The cortical model was especially constructed using 2.6 million spiking neurons and 5.3 billion synaptic connections based on DTI data. They showed that biologically reasonable whole-body movement and cortical dynamics emerge from the interaction among the brain, body, and environment.

**Fig 1:**
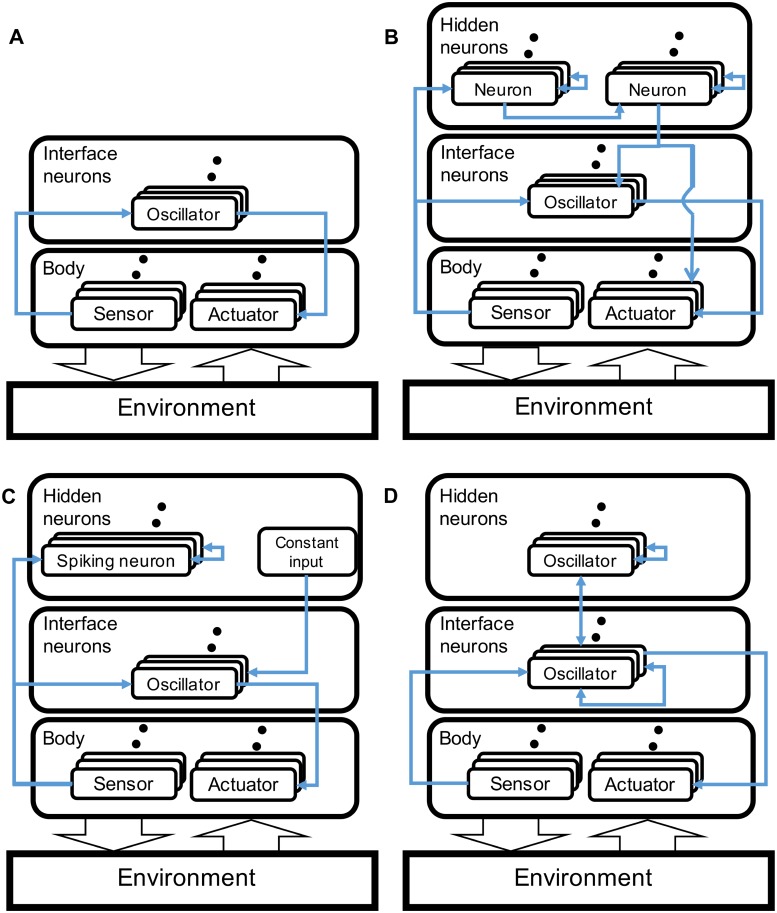
Differences in the network structure of the proposed model from the existing studies. (A) No connectivity is found between the interface neurons [32, 34]. Furthermore, the hidden neurons, which are supposed to be a cortex to represent brain dynamics, are missing. (B) Hidden neurons are observed, but still no connectivity is found between the interface neurons [33]. (C) Constant input from the hidden neurons is provided to the interface neurons, no influence from hidden neurons to body to generate motor behavior [35]. (D) A model in this study. Fully wired connections among neurons in the brain.

As has been mentioned in the previous section, wired and information networks have been thought of as important factors to understand the mechanism for the emergence of behavior and its transition, which are not considered in the abovementioned studies. To deal with this problem, the model needs to have a structure to express the coupled dynamics of the brain and the body. However, the abovementioned models have been realized only partially, that is, missing some parts. Fig 1 shows the differences in the network structure of the propose model from these existing studies. In these studies [32, 34], there is no connectivity exists between the interface neurons. Furthermore, hidden neurons, which are supposed to be a cortex to represent brain dynamics, are missing (Fig 1(A)). Hidden neurons are found, but still no connectivity is observed between the interface neurons in [33] (Fig 1(B)). In the case of a detailed fetal simulation [35], the constant input from the hidden neurons is provided to the interface neurons, which means no influence from the hidden neurons to the body to generate a motor behavior (Fig 1(C)). The neural architectures in [33, 35] consider the biological brain structure. Therefore, the difference between the interface and the hidden neurons is not clear, which could making the network analysis behind the emerged behavior intractable because of complicated connections among neurons. This is a reason why they have not addressed this issue. Compared to them, our model has a clear difference between the interface and the hidden neurons and in the interactions at both layers to make the network analysis tractable and informative. Instead, a simple body structure (i.e., a snake-like robot) is adopted to realize a tractable analysis of the coupled dynamics between a physical body and a brain network, which the previous studies have not addressed.

The main objective of this article is to comprehend the relationship between information network and the emergence of behavior, which occurs through the interaction among wired networks (neural architecture) and the body (musculoskeletal model) in an environment. We suppose that the number of emerged behaviors is affected by the topological property of wired networks and the degree of influence between wired networks and the body. These emerging behaviors are characterized as stable and unstable movement patterns based on the relative stability (duration of behavior). Therefore, we assume these stable and unstable movement patterns corresponding to a behavioral attractor and the transition between attractors, respectively. We hypothesize that different interactions and topological properties in an information network induce stable or unstable movement patterns. Herein, the information network is estimated based on the causality between the neurons in a wired network.

The main procedures and analysis are presented as follows:

1. construction of a nonlinear oscillator network (wired network) with a musculoskeletal system to generate spontaneous movement patterns and its transition based on the dynamical system approach;
2. understanding the role of the wired network to understand the emergence of both behavior and information network by varying the topological network structures of the wired network;
3. clustering of emerging behaviors based on the correlation of joint angles of the musculoskeletal system; and
4. analyzing the network structure corresponding to the classified behavior, utilizing transfer entropy and complex network theory, and focusing on sub-network connections among them.

## Musculoskeletal model and complex network with nonlinear oscillator

### Network with a nonlinear oscillator

**Fig 2:**
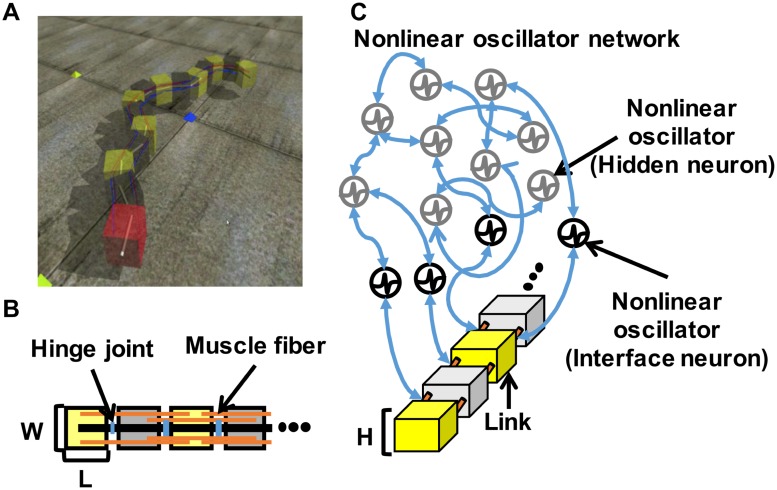
Snake-like robot with a nonlinear oscillator network. (A) Snake-like robot. (B) Physical model of the snake-like robot. (C) Schematic of the snake-like robot with a nonlinear oscillator network. The snake-like robot consists of multiple links connected via muscle fibers and hinge joints. The nonlinear oscillator network consists of hidden (gray node) and interface neurons (black node) that are indirectly and directly connected to the snake-like robot muscle fibers, respectively.

We conducted a physical simulation using Open Dynamics Engine (ODE) [63] with a snake-like robot and a nonlinear oscillator network (Fig 2).

### Nonlinear oscillator network

In this study, the synchronized and desynchronized dynamics between the rhythmic activities of the brain region described in the “Wired and information network structures in the brain” section is expressed by using a nonlinear oscillator network. The nonlinear oscillator network consists of using multiple nonlinear oscillators connected by a complex network model. The network and the musculoskeletal models are mutually connected through motor commands and sensory signals, and the interaction generates rhythmic neural and bodily behaviors. The oscillators are separated into “interface” and “hidden” neurons. “Interface” neurons provide motor commands, receive sensory signals, and interact with other neurons. “Hidden” neurons only interact with other neurons (Fig 2). The interface neurons are directly connected to the robot muscles, receive sensory feedback on muscle lengths, and send signals to adjust the length of muscle length. The hidden neurons are indirectly connected to the musculoskeletal model, and their activations affect and are also affected by the interface neurons and other hidden neurons. In this study, we employed the Bonhoeffer-van der Pol (BVP) equation to model the activity of each oscillator (Eqs (1) and (2) below). The interactions between the oscillators are modeled by using Eq (3). The interactions between multiple BVP oscillators (Eqs (1)–(3)) induce periodical and non-periodical complex behaviors depending on the interaction parameters [64].

$$\begin{array}{c} \tau \frac{dx}{dt}=c\left(x-\frac{1}{3}x^{3}-y+z\right)+\delta \left(S_{f}-x\right), \end{array}$$ (1)

$$\begin{array}{c} \tau \frac{dy}{dt}=\frac{1}{c}\left(x-by+a\right)+\epsilon S_{f}, \end{array}$$ (2)

$$S_{f}=\{\begin{array}{ll} \alpha I+\left(1-\alpha \right)\frac{1}{K}\sum_{j=1,j\neq i}^{N}w_{ji}x_{j} & \text{if interface neuron},\text{ or}\\ \frac{1}{K}\sum_{j=1,j\neq i}^{N}w_{ji}x_{j} & \text{else}. \end{array}$$ (3)

Each neuron has a connection weight *w*, and *K* represents the number of connections for each neuron. In the above equations, *a*, *b*, and *c* control the neuron behavior; *z* is a tonic input; and *δ* and *ϵ* control the strength of the excitatory and inhibitory influences from other neurons, respectively. Finally, *α* controls the strength of the ratio between the body and the network, while *I* is the sensory feedback value from the musculoskeletal model. The movement of the robot is generated by a spontaneous activation of the non-linear oscillator network if *α* = 0.0. Each interface neuron is independently activated using the sensory feedback from the body only if *α* = 1.0. In our simulation, we set *a* = 0.7, *b* = −0.2, *c* = 2.0, *δ* = 0.01, *ϵ* = 0.015, *α* = [0.0, 1.0], and *z* = 0.4, 0.45, 0.5 or 0.55. The network includes 26 interfaces and 174 hidden neurons. We utilized the following weight types for *w*:

- Uniform weights: $w_{j,i}=\{\begin{array}{cc} 1 & \;\text{if}\;j,i\;are\;connected,or\\ 0 & \;\text{else}\; \end{array}$
- Randomly distributed weights: the weights are randomly distributed in [−1.0; 1.0] and normalized to $\sum_{i=1,i\neq j}^{N}|w_{i}|=1$.

### Complex networks

A complex network has connections that are neither purely regular nor purely random. Small-world and scale-free networks are typical complex networks.

#### Small-world network

In the small-world network proposed by Watts and Strogatz (WS) [41], the shortest path length is defined as the number of steps to pass from one node to another compared to the total number of nodes in the network. A small-world network can be generated by rewiring a regular network. The algorithm to generate a small-world network is presented as follows:

1. Begin with a regular network, where each node is connected to the *m* nearest nodes to create a ring pattern.
2. Rewire each node connection randomly according to the rewiring probability *p*. The network is purely regular if the probability *p* = 0 and purely random if *p* = 1. The value of *p* for a small-world network is typically between 0.01 and 0.1.

#### Scale-free network

A scale-free network is defined as a network in which the number of node connections follows the power-law distribution given by *P*(*k*)∼*k*^ − *γ*^, where *k* is the number of connections for each node, and *γ* is typically between 2 and 3. In this study, we constructed a scale-free network based on the Barabasi-Alber (BA) [42] model as follows:

1. Begin with an initial number of nodes (*m*_0_).
2. Add a new node with *m*(< *m*_0_) connections to the already existing nodes with probability *P*(*k*_*i*_), where
  $$\begin{array}{c} P\left(k_{i}\right)=\frac{k_{i}+1}{\sum_{k}k_{j}+1} \end{array}$$ (4)
3. Repeat Step 2 until the prespecified number of nodes is added.

### Network structures of nonlinear oscillators

We utilized the following network types and parameters of the nonlinear oscillator networks in our experiments.

1. Regular network: *m* = 2;
2. Small world network (WS model): *p* = 0.01 or 0.05;
3. Random network (based on WS model): *p* = 1.0; and
4. Scale-free network (BA model): *m*_0_ = 2.

The following complex network properties are considered to understand the topological characteristics of the network:

- Average clustering coefficient: The degree of clustering based on the number of closed triads consisting of any combinations of triplets each of which consists of three connected nodes in the network [65]. The clustering coefficient for a weighted directed network is defined as follows:
  $$\begin{array}{c} C_{i}=\frac{{\left[W^{\left[1/3\right]}+W^{T^{\left[1/3\right]}}\right]}_{ii}^{3}}{2\left[k_{i}^{tot}\left(k_{i}^{tot}-1\right)-2k_{i}^{↔}\right]}, \end{array}$$ (5)
  $$\begin{array}{c} k_{i}^{tot}=k_{i}^{in}+k_{i}^{out}={\left(A^{T}+A\right)}_{i}1, \end{array}$$ (6)
  $$\begin{array}{c} k^{↔}=\sum_{j\neq i}a_{ij}a_{ij}=A_{ii}^{2}, \end{array}$$ (7)
  $$\begin{array}{c} \bar{C}=\frac{1}{N}\sum_{i}^{N}C_{i} \end{array}$$ (8)
  where **W** is a weight matrix; **A** is a connection matrix; and *k*^*tot*^, *k*^*in*^, and *k*^*out*^ are total degree, in-, and out-degree, respectively. A larger coefficient indicates that the more nodes are interacting with each other in the same group of the network.
- Average shortest path length: the shortest number of steps necessary to pass from one node to another [66]. The shortest path length is defined as follows:
  $$\begin{array}{c} a=\sum_{i\neq j}\frac{d(i,j)}{n(n-1)}, \end{array}$$ (9)
  where *d*(*i*, *j*) is the shortest path from a node *i* to *j*, and *n* denotes the number of nodes in a network. Here, we use the inverse value of weight as a distance between nodes. Therefore, a larger value of weight indicates that the distance between nodes is less. A smaller average shortest path length indicates that the network is connected more globally.

**Fig 3:**
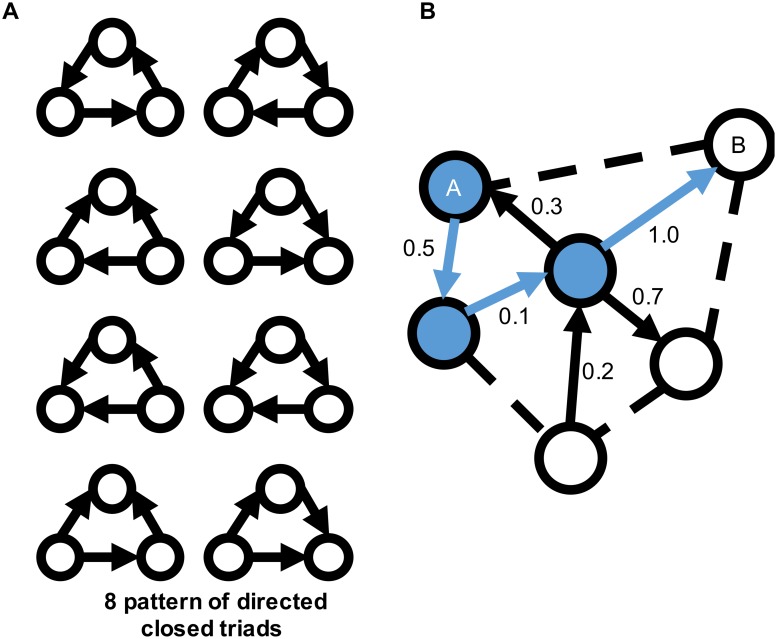
Examples of a clustering coefficient and the shortest path length. (A) Eight different patterns of directed closed triads to calculate the clustering coefficient: The clustering coefficient denotes the percentage of the existence of eight patterns of closed triads in a network. (B) Example of closed triads in a network and shortest path: the black arrows and the black dashed lines represent the directed connections between nodes and possible connections, respectively. Each number on the arrows denotes the weight between nodes. The blue arrow shows the shortest path from node A to node B, while the blue nodes are closed triads to calculate the clustering coefficient of a network. We consider the value of weights to calculate the clustering coefficient and the shortest path length.

**Fig 4:**
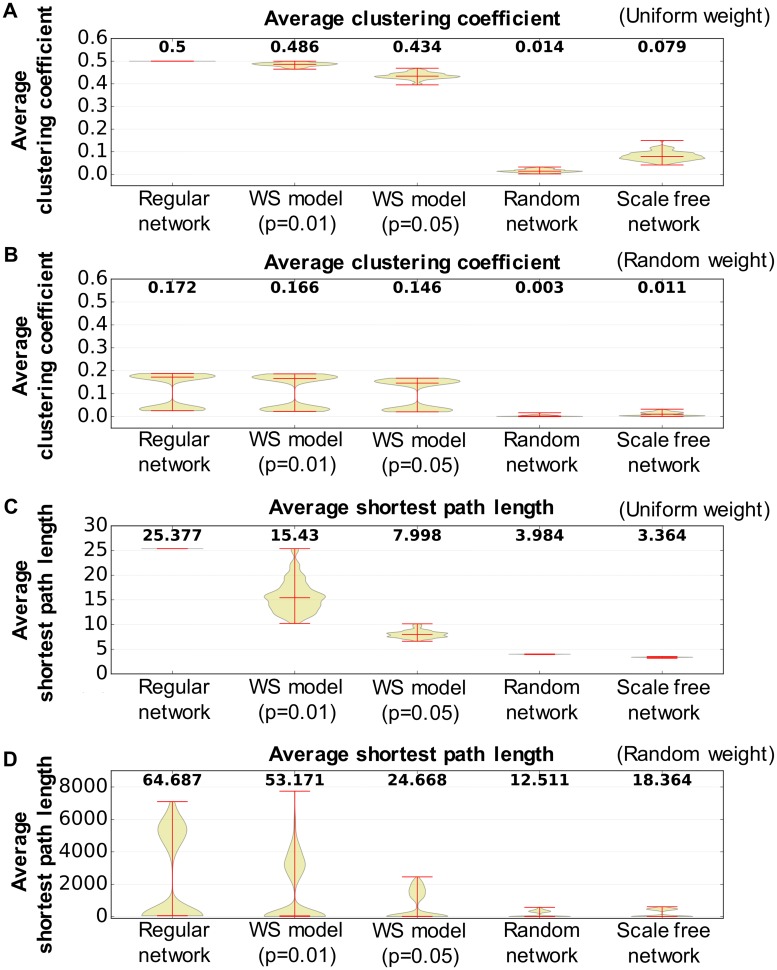
Complex network properties of wired networks employed in the experiments. The number in each box plot denotes the value of the median of 100 measurements for different experimental settings for each network type. (A) Average clustering coefficient for the uniform weights. (B) Average clustering coefficient for the randomly distributed weights. (C) Average shortest path length for the uniform weights. (D) Average shortest path length for the randomly distributed weights.

**Fig 5:**
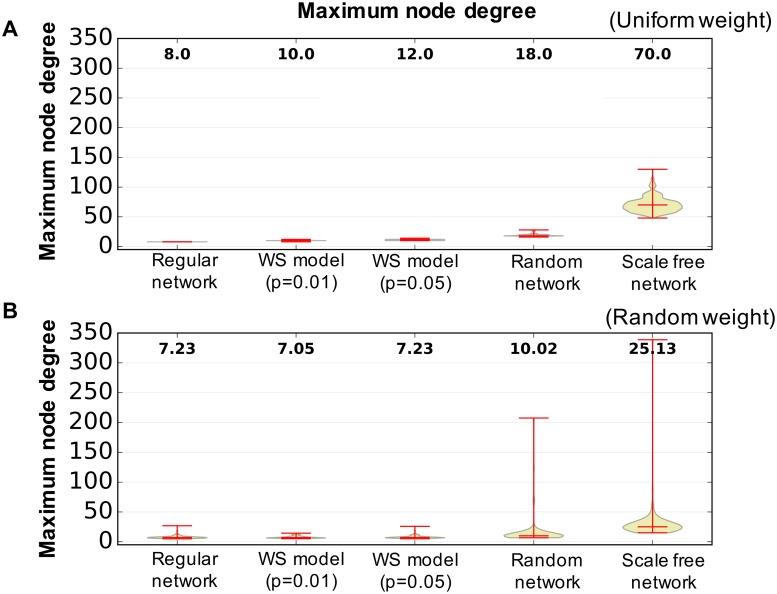
Maximum node degree of the wired network employed in the experiments. The number in each box plot denotes the value of the median of 100 measurements for different experimental settings for each network type. (A) Uniform weights and (B) randomly distributed weights.

Fig 3 shows examples of closed triads to calculate the clustering coefficient and the shortest path length in a network. We assume that information is transmitted from one node to another, regardless of whether the weight between the nodes has a positive or negative value. Hence, we use the absolute value of weights to calculate the clustering coefficient and the shortest path length. Complex networks typically have a higher clustering coefficient and a smaller shortest path length. Fig 4 shows the clustering coefficient and shortest path length of each nonlinear oscillator network in our experiments. As shown in the figure, a randomly distributed weighted network shows a higher shortest path length and a smaller clustering value, implying that randomly distributed weighted networks require more time to transmit information from one node to another node using less interaction among the neighboring nodes. Fig 5 presents the maximum node degree of each nonlinear oscillator network. The node degree herein is calculated by $degree_{i}=\sum_{i\neq j}^{N}|w_{j,i}|$. A larger value implies that the network has a hub node, which has more degrees than other nodes. Several studies have showed that this hub node plays an important role in maintaining the connectivity of the network [67] and efficiently transferring information [68]. We expect a difference in information transmission because such a structural difference affects the stability of the movement pattern in chaotic itinerancy.

### Snake-like robot

We used the snake-like robot in our simulation because of the following reasons:

1. We need a simplified body, which is not so complicated as a fetal body that may have too many parameters to specify, to make the analysis of the relationship between the emerged behavior and the network structure behind it tractable.
2. We need a complex body to have behavior variations sufficient for the analysis to be rational and informative.

Between (1) and (2), we decided to adopt a snake-like robot as a physical body. We constructed a model of the snake-like robot using ODE [63]. Fig 2 shows the model and the appearance of the snake-like robot. The robot is designed to exhibit synchronized movement patterns using two-joint muscles between the body links. These links are connected using hinge joints and muscle fibers. Each muscle fiber is stretched and compressed to move the robot body based on the muscle model shown in [69]. The muscles are connected to the interface neurons that receive the muscle lengths as sensory feedback values. Moreover, we restrict the movement in two dimensions through a hinge joint to simplify the analysis of the movement patterns and its interpretation. The parameters of this snake-like robot are shown in Table 1 shows the parameters of this snake-like robot.

**Table 1:** Setting of the snake-like robot.

Link height	Link width	Link length	Gap between two bodies
0.1 [m]	0.1 [m]	0.1 [m]	0.02 [m]
Link mass	Number of links	No. of single-joint muscles	No. of double-joint muscles
0.6 [kg]	15	0	26

### Experimental settings

The experiments were conducted using the above-mentioned network structures, and the sensor ratio *α* in Eq (3) was varied as [0.0, 0.1, 0.3, 0.5, 0.7, 0.9, 1.0]. The tonic input was varied as [0.4, 0.45, 0.5, 0.55]. In this study, we conducted 100 experiments for each condition, and the simulation time was 2000 s for each condition. We excluded the first 50 s from the analysis because of too much unstable movement of the snake-like robot.

## Behavior and network analyses

We applied the following procedures to cluster and classify the emerging movements and understand the relationship between the movement and its network topology:

### Behavior analysis

We estimated the correlation coefficients between the hinge joint angles within a time window, from which these feature vectors were extracted. We then applied a clustering method to the feature vectors to distinguish between the repetitive movement patterns. The procedure is presented as follows:

1. Calculate a feature vector that consists of correlation coefficients for all possible joint angle combinations in terms of window positions with time shifts. This vector is calculated as follows:
  $$\begin{array}{c} R=\left[r^{1,2},r^{1,3},\cdots r^{1,k},r^{2,3},r^{2,4},\ldots ,r^{k,k-1}\right], \end{array}$$ (10)
  $$\begin{array}{c} r^{i,j}=\left[r_{1}^{i,j},r_{2}^{i,j},\ldots ,r_{n-1}^{i,j},r_{n}^{i,j}\right], \end{array}$$ (11)
  where *r* is a correlation coefficient; *k* is the number of joint angles; and *i* and *j* are the indices of the joint angles.
  $$\begin{array}{c} r_{n}^{i,j}=\frac{\sum_{l=n*t_{s}}^{t+\Delta t}\left(\theta_{l}^{i}-{\bar{\theta }}^{i}\right)\left(\theta_{l}^{j}-{\bar{\theta }}^{j}\right)}{\sqrt{\sum_{l=n*t_{s}}^{t+\Delta t}\left(\theta_{l}^{i}-{\bar{\theta }}^{i}\right)}\sqrt{\sum_{l=n*t_{s}}^{t+\Delta t}\left(\theta_{l}^{j}-{\bar{\theta }}^{j}\right)}}, \end{array}$$ (12)
  where *θ* is the joint angle; $\bar{\theta }$ is the mean of the joint angles in a time window; *n* is an index of the window position; and Δ*t* and *t*_*s*_ denote the sizes of the time window and the shifting time, respectively.
2. Reduce the number of the dimensions of the feature vectors by applying the Laplacian eigenmaps for clustering [70]. Laplacian eigenmaps are manifold unsupervised learning algorithms for non-linear dimension reduction, which projects each sample point into a low-dimensional space based on Laplacian eigenmaps to retain the local geometric properties in the k-nearest neighbor points for each point.
3. Find clusters using the density-based spatial clustering of applications with noise (DBSCAN) [71]. DBSCAN can determine the arbitrary number of clusters with arbitrary shapes based on the density of a given set of points in space. This algorithm considers it as one cluster if the distance between the data is less than a parameter *ε*, and the number of data points is more than a minimum number of points.
4. Measure the duration of each movement pattern and classify the patterns based on whether the durations are shorter or longer than a threshold value, which is determined by the Otsu method [72]. Hereafter, we classify these movement patterns as unstable (less than the threshold) or stable (longer than the threshold).

### Movement of the snake-like robot

**Fig 6:**
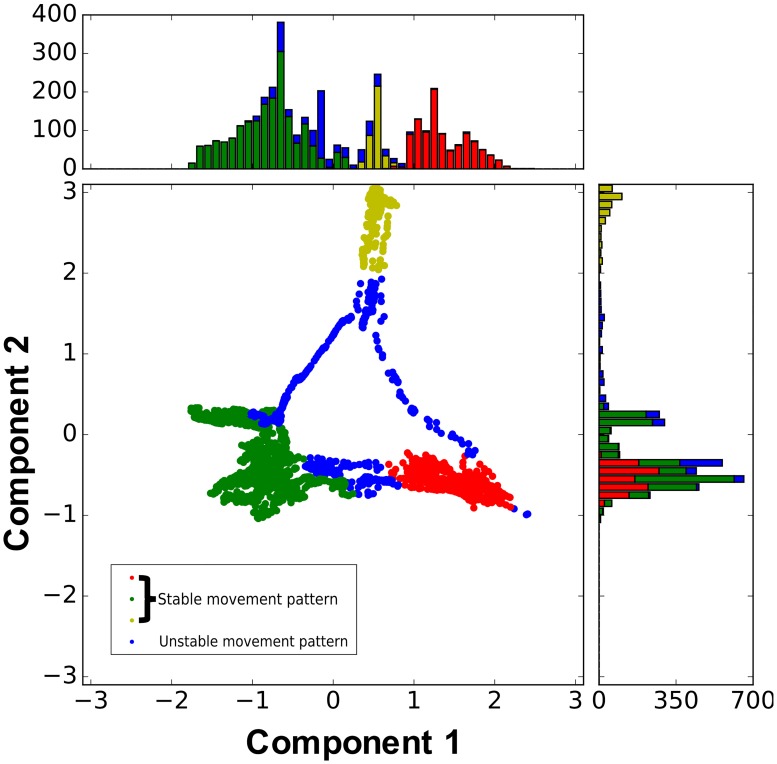
Example of the feature vector in the dimensionality-reduced space by Laplacian eigenmaps (WS model (*p* = 0.05) with randomly distributed weights, a tonic input of 0.4, and a sensor ratio of 0.3). The blue dots represent an unstable movement pattern. The dots with other colors represent stable movement patterns. The bar graphs show the histogram of data points along the x and y axes.

More than four different stable movement patterns were observed in the simulation: crawling movement (forward crawling, backward crawling) and bending (hold a position, sidewinding). These movement patterns and their transitions emerged at different durations. The state of the robot in the low-dimensional space is changes with time among the clusters that correspond to one movement pattern, like chaotic itinerancy. See Supporting information S1 Video for an example of the changes of the state of the robot and the movement pattern with time. We reduced the 91 dimensions of the feature vector to three dimensions using 350 k-nearest points as shown in (Fig 6). After dimensionality reduction, we clustered each point with *ε* = 0.16, and the minimum number of points was set to 10.

### Relationship between various movements and the nonlinear oscillator network

**Fig 7:**
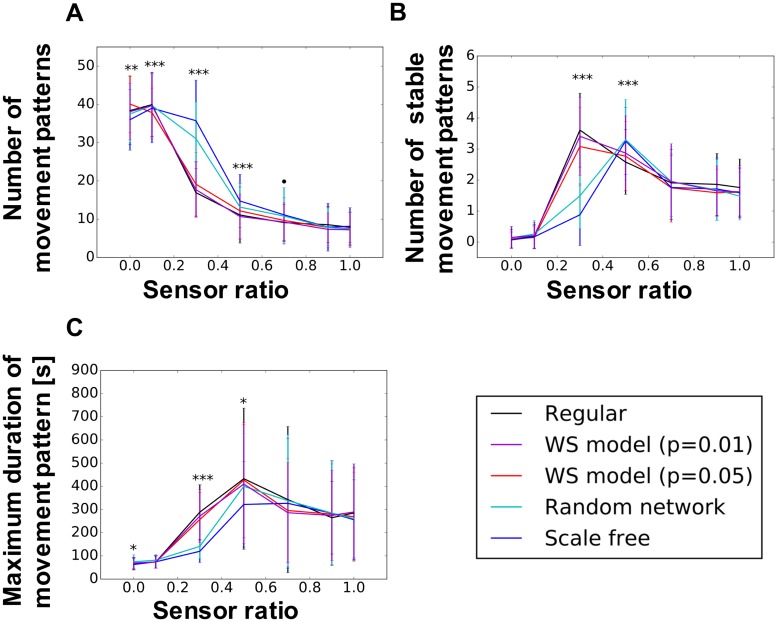
Number of movement pattern and maximum duration of movement for the uniform weight. The tonic input is 0.45. (A) Number of movement patterns, (B) number of stable movement patterns, and (C) maximum duration of movement patterns. The x-axis indicates the sensor ratio necessary *α* in Eq (3) to control the proportional influences between the body and the network. *** *p* < 0.001, ** *p* < 0.01, * *p* < 0.05, ⋅ *p* < 0.1 indicate statistically significant differences between the wired networks through the ANOVA test.

**Fig 8:**
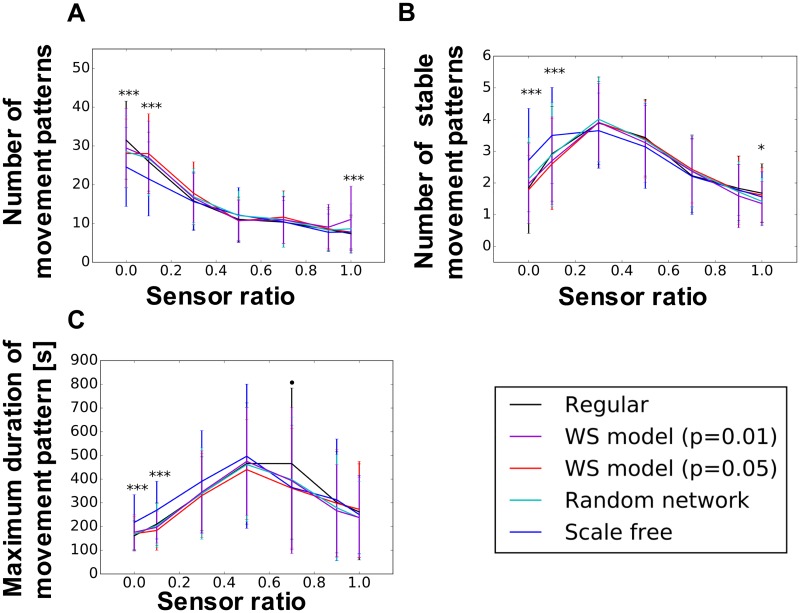
Number of movement pattern and maximum duration of movement for the randomly distributed weight. The tonic input is 0.45. (A) Number of movement patterns, (B) number of stable movement patterns, and (C) maximum duration of movement patterns. The x-axis indicates the sensor ratio necessary *α* in Eq (3) to control the proportional influences between the body and the network. *** *p* < 0.001, ** *p* < 0.01, * *p* < 0.05, ⋅ *p* < 0.1 indicate a statistically significant differences between the wired networks through the ANOVA test.

Figs 7 and 8 show the average number of movement patterns, average number of stable movement patterns, and the maximum duration of one movement pattern in each network structure for the uniform and randomly distributed weights, respectively. We show herein the results when the tonic input is 0.45, because there are no big differences from the different value of tonic input (see S1–S6 Figs for the effect of the tonic input). For this study, the threshold used to classify the movement pattern as stable or unstable is determined as 101.5 s using the Otsu method. The x-axis indicates the sensor ratio *α* in Eq (3). As shown in the figures, different peak patterns appear depending on the network type and the tonic input.

As shown in (a) of Figs 7 and 8, the number of movement patterns shows a tendency to decrease along the x-axis. Therefore, the strong feedback from the bodily movements constrain the neural activity, and the movement patterns are reduced. However, as shown in (a) of Figs 7 and 8, the majority of the peaks are positioned near the center of the sensor ratio range, whereas only a small number of movement patterns can be observed in the vicinity of 1.0 and 0.0. Furthermore, the result of the maximum duration of the movement pattern in (c) of Figs 7 and 8 shows that more stable movement patterns are observed when the value of the sensor ratio is high.

These results imply that a higher number of movement patterns emerge by adding the dynamics of the network. However, a too strong dynamics of the network causes the loose of the stability to sustain the current behavior. This may imply that a balance between the wired networks and the body of the robot is important for diverse behaviors to emerge. Statistically significant differences exist in terms of the network type.

### Analysis of the network dynamics

We estimate the information network for each movement pattern identified in the behavioral analysis to understand the dynamic properties of the neural network during behavior emergence. In this study, we used transfer entropy to estimate an information network to consider the direction of the information flow between the neurons or the brain (nonlinear oscillator)-body (musculoskeletal model) because in the interaction between the neurons or brain-body, it is considered that one might initiatively send an information to another. The transfer entropy (TE) from one neuron y to another neuron x is given as follows:

$$\begin{array}{c} T_{y\to x}=\sum p(x_{n+1},x_{n}^{\left(k\right)},y_{n}^{\left(l\right)})\log\frac{p(x_{n+1}|x_{n}^{\left(k\right)},y_{n}^{\left(l\right)})}{p(x_{n+1}|x_{n}^{\left(k\right)})}, \end{array}$$ (13)

where *l* and *k* denote the given historical lengths used to predict the future state and *t* indicates the current time step. In this study, *l* = *k* = 1. The Java Information Dynamics Toolkit [73] is used to calculate the TE using the Kraskov, Stogbauer, and Grassberger (KSG) method [74]. Note that the KSG method has a greater accuracy for a smaller number of samples compared to other methods. The resultant information network models show that the causal relationship between the neurons and the network structure varies depending on the movement patterns. The extracted information network structure is then analyzed to answer the following three questions: (1) What is the nature of the spatial interactions between the neurons (local or global)? (2) How complex is the network structure (high or low complexity)? (3) How strongly are the neurons connected to the environment through the body?

The procedures used to answer to these questions are as follows:

1. Apply the infinite relational model (IRM) [75] to visualize the information network structure.
  - Estimate mutual information to extract subnetworks, such as brain regions.
  - Binarize the result of (a) to apply the IRM because it can only handle binary values. The value of the threshold to be binarized is determined by the Otsu method [72].
  - Rearranges a matrix consisting of relational data variables to a diagonal sequence of submatrices, which may correspond to the subnetworks.
  - Overlay TE values on the result of (c) to extract the final subnetwork structure.
  Eq (1) in [75] includes the hyper-parameters *γ* and *β*, which denote the frequency of the new clustered created using the Dirichlet process and the noise of the relationship among the elements in a sub-cluster, respectively. We set *γ* = 1 and *β* = 7.
2. Calculate the complex network properties for the information network to determine the global properties of the network
3. Calculate the average of the two TEs from (to) the hidden neurons to (from) interface ones understand the relationship between the robot body and the information network.

### Analysis of the information network

**Fig 9:**
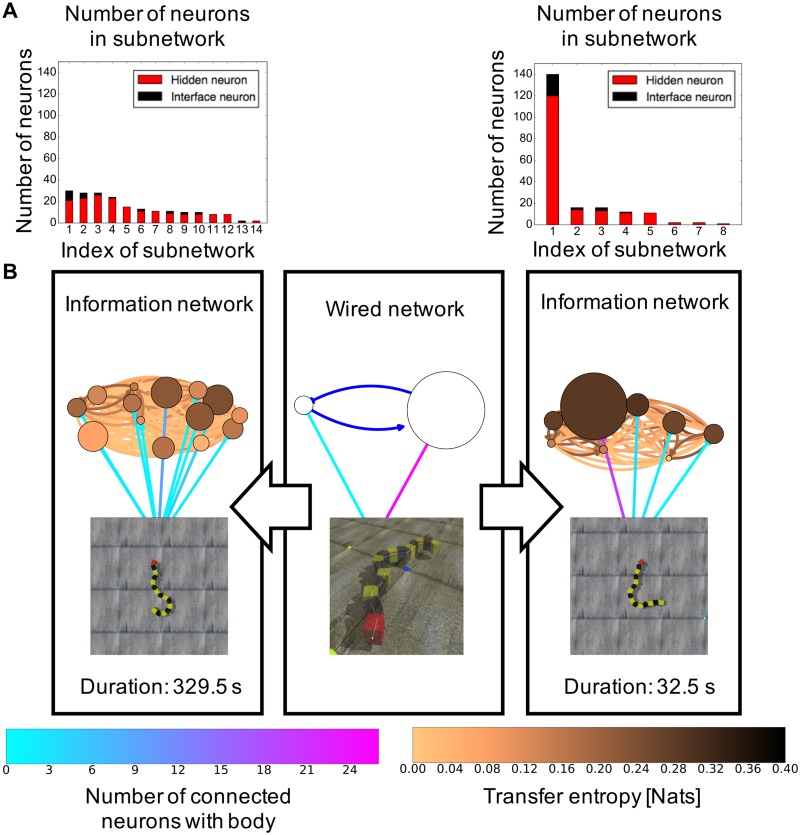
Estimated information network structures for different movement durations: 329.5 s (longer) and 32.5 s (shorter). (A) Number of neurons in each subnetwork for 329.5 s and 32.5 s, respectively. The red and black bars indicate hidden and interface neurons. (B) Wired network with a musculoskeletal movement and two different information networks. Each node indicates an IRM-extracted subnetwork, and the node sizes indicate the number of neurons in each subnetwork.

Fig 9 shows the topological difference between a wired network and two information networks corresponding to two different behaviors in one simulation. As shown in the figure, different network topologies emerged from the interaction, even though the structure of the wired network is fixed. Moreover, two information networks differ in two aspects. A longer movement pattern has smaller subnetworks (node) with less communication among them (bright cooper and black links between nodes) than a shorter movement pattern. Furthermore, the interface neurons are sparsely distributed in the subnetworks (cyan and magenta links between the body). Meanwhile, the shorter movement pattern has an information network consisting of several small subnetworks and one big subnetwork that includes more interface neurons than the other subnetworks.

**Fig 10:**
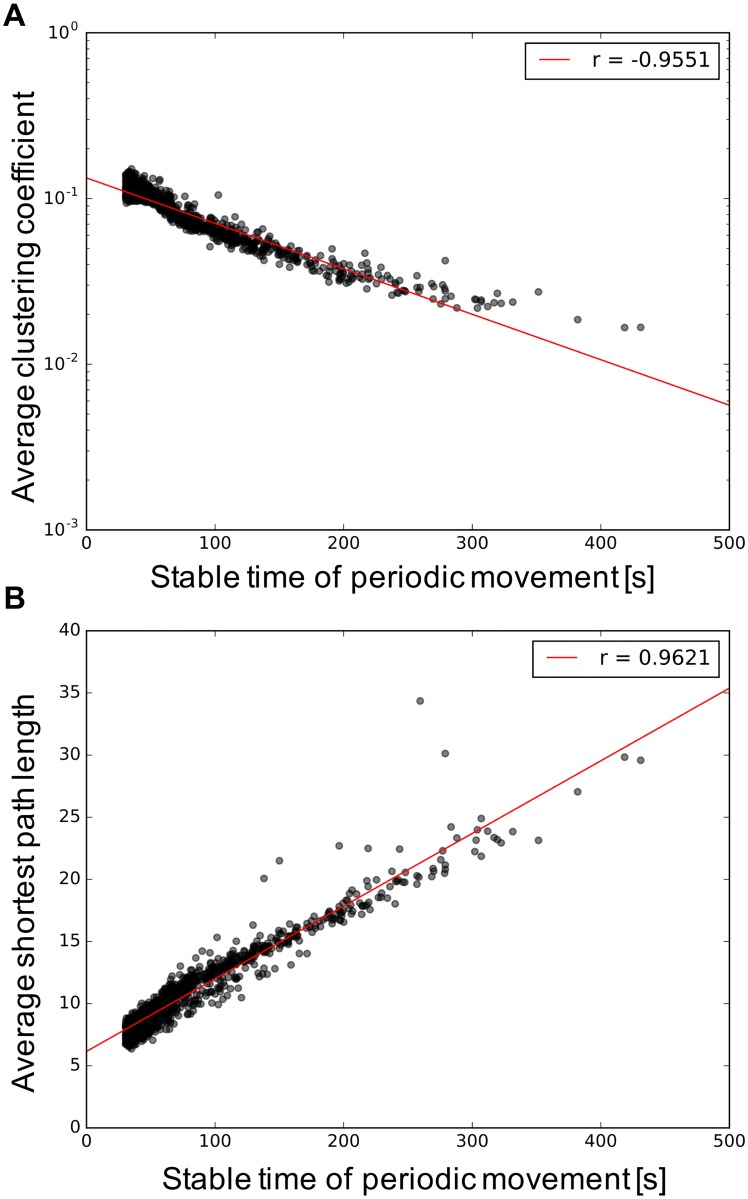
Structural property of the information network for the uniform weights. Each red line in the figure indicates correlation. (A) Average clustering coefficient and (B) average shortest path length.

**Fig 11:**
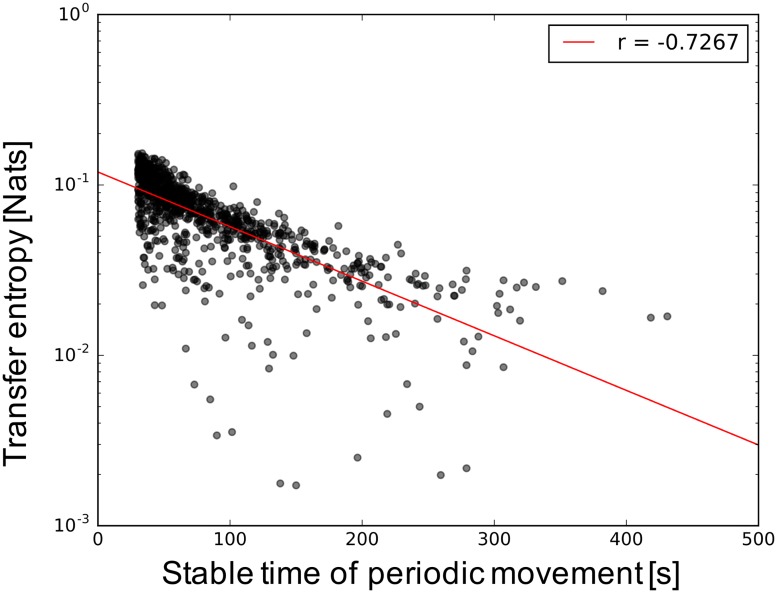
Influence between the body and the network in terms of the duration of periodic movements for the uniform weights. The red line in the figure indicates a correlation: average of transfer entropy from the interface neurons to hidden neurons.

Figs 10 and 11 show the structure properties of the information network and the interaction between the interface and hidden neurons in terms of the sustainability of the periodic movement. No differences exist among different types of the wired networks, here, we plot results regardless of structure of wired network with a uniform weight. These results without difference might indicate that a common information structure not affected by the structure of the wired network is related to the duration of the movement pattern, unlike the number of the emergence of behaviors, which is affected by the structure of the wired network. Fig 10 shows the properties of the clustering coefficient and the shortest path length of the information network in terms of the sustainability of the periodic movement regardless of the structure of the wired network. For longer-duration movement patterns, it is apparent that the information network has a positive correlation with the shortest path length and a negative correlation with the clustering coefficient. A high value of the shortest path length and a small value of the clustering coefficient in the network is typically observed in a network that does not have complex network properties. Fig 11 shows the transfer entropy from the hidden to the interface neurons regardless of the structure of the wired network as a means of determining the influence of the environment on the network through the robot body. We only plot the hidden to the interface neurons because no differences are found between hidden to interface and interface to hidden. As seen in this figure, the transfer entropy between the interface and hidden neurons decreases in accordance with the duration of the movement pattern. The above results indicate that a part of the subnetworks contributes to the stable movement patterns and the interaction vigorous interaction with many hidden neurons, which induces a transition of the current movement pattern to another movement pattern.

## Discussion

### Role of the body and brain dynamics in bodily chaotic itinerancy

We have shown the emergence of movement patterns and their transitions as a consequence of the interactions between a musculoskeletal system and nonlinear oscillator networks. Figs 7 and 8 illustrate how the number of (stable) movements and the duration of the most stable movement change with respect to the sensor ration alpha and the type of the wired network structure. As shown in (a) and (b) of Figs 7 and 8, many movement patterns with a short duration emerged when the sensor ratio is 0.0. Therefore, the movements of the snake-like robot are spontaneously generated by the network itself without sensory feedback from the body. However, the number of emerged movement patterns with a short duration decreases, while the stable movement pattern increases along the increasing sensor ratio. Therefore, the influence of the body dynamics increases. These results indicate that the dynamics in the network generates various random and unstable movement patterns. However, the dynamics itself cannot stabilize the movements because of the strong chaotic dynamics of the network. Meanwhile, the dynamics of the body may provide mechanical stability to maintain the current movement pattern. The maximum duration of the movement pattern shown in (c) of Figs 7 and 8 also supports this. However, the movement pattern decreases if the sensor ratio exceeds 0.5. We consider that weak attractors are formed around a strong attractor because synchronicity is weakened by loose connections between neurons if the sensor ratio is high, thereby making it difficult for the robot to converge to one movement pattern. The interpretation of the role of the body and the brain for the emergence of behaviors might be related to the study of spontaneous activation in the neocortex [76]. The authors showed that the activation pattern for encoding sensor information is already shown in the spontaneous activations of neocortical activities, and its activation is constrained by sensory input through experiment using a rat. Therefore, the sensory feedback may be a trigger to self-organize a pattern of bodily behavior into a subspace in one of the possible states determined by a non-linear oscillator network.

### Network structure of information network behind the classified behavior

Figs 9 and 10 show that more unstable movement patterns emerge when the neurons in an information network are actively interacting using one big subnetwork with a large value of the clustering coefficient and the shortest path length. This suggests that the snake-like robot tried to more efficiently explore a stable movement pattern or transit another movement through an information network with a small value of the shortest path length and a large value of the clustering coefficient. The speed of information transmission is fast, and the frequency of interaction is high. Meanwhile, in the presence of a stable movement pattern, the interface neurons are distributed in subnetworks that locally interact with the body, and the information network has less complex network properties. The transfer entropy in the case of stable movement patterns is low as shown in Fig 11, which also supports this discussion. These positive and negative relationships between the structural property of the network and the duration of movement pattern indicate that we can conversely infer the probability of transmitting from the current behavior to a different behavior by examining the current structure of the information network. This finding can suggest a mechanism of chaotic itinerancy in terms of the animal’s spontaneous activities from the aspects of neural activities, such as a default mode network [77], which is a network consisting of activation signals among several brain areas of the cortex when a person is in a resting state. Hermundstad et al. [6] showed that the shortest path length decreased when the task to be concentrated on is given. Therefore, we speculate the information network for stable and unstable movement patterns corresponding to unfocused (unconscious) and focused states (conscious).

### Influence of the network types onto the number and duration of movement patterns

Figs 7 and 8 shows statistically significant differences in terms of a wired network type. We suppose that this difference is caused by the distribution of nodes in a wired network. As shown in Fig 5, a scale-free network presents a large difference in the maximum node degree. Therefore, a scale-free network has a hub node with a larger node degree than the other nodes. Several studies showed that this hub node plays an important role in maintaining the connectivity of the network [67] and in efficiently transferring information [68]. In this regard, this hub node may induce different dynamics of the model. Furthermore, whether the hub nodes consist of interface or hidden neurons may also influence the model dynamics. Nevertheless, this result indicates that the type of wired network may influence the emergence of diverse behaviors. Moreover, differences can be found between the uniform and randomly distributed weights. This result might relate with a similar interpretation of the information network. Fig 4 shows a randomly distributed weighted network with a larger shortest path length and a small clustering coefficient, which means that the network requires more time to transmit information from one node to another one and less interaction among the neighboring nodes. Such properties of the wired network correspond to the properties of the information network, which shows a longer duration of the movement patterns. We suppose that these dynamics for the transition of the state and the network properties of the wired network make a difference between randomly distributed and uniform networks (Figs 7 and 8).

These results show that the information structure has a common structure property to the stability of the movement pattern regardless of the structure of the wired network, but the frequency of the appearing movement pattern is different depending on the sensor ratio and the structural property of the wired network. This result might support the existing argument that the formation of the connectivity between the body and brain dynamics by subplate is important for the emergence of the diverse behavior as has been explained in the “Diverse behavior and network” section, and show how the structural property of the wired network affects the coupled dynamics. Therefore, a balance of the two dynamics of the body and the network determines the property of the chaotic itinerancy of the behavioral attractors of the coupled system. If either side of the coupled dynamics dominates other dynamics, the diversity of the behaviors disappears, but their tendency to increase the number of emerged movements along with the sensor ratio is different with respect to the structure of the non-linear oscillator networks, especially the distribution of nodes. Moreover, the general movement, which is a diverse movement pattern in early childhood, and the change in diverse movement patterns with age [78] may also be related with this finding in terms of behavioral dynamics. More analyses are needed to verify the abovementioned speculations.

### Future issues

Many issues need to be addressed. First, a relationship between a subnetwork and the behavior is not clear. Even though we showed that a subnetwork with interface neurons locally interacts with the body in the case of a stable movement pattern, the roles of other subnetworks are not clear. As we have mentioned earlier from the fMRI studies, each local network in the brain has a variable connectivity to perform different tasks [4]. Cole et al. [2] showed that the fronto-parietal brain network acts as a hub region in a wide variety of connectivity and could be used to identify the current task and to transit another state from to switch to a novel task. We expect some subnetworks in our simulation to act as a hub or play a different role, such as inhibiting or suppressing the activation of other subnetworks. Furthermore, the result of this research shows that a different information network with subnetworks emerges corresponding to each movement pattern in a bodily chaotic itinerancy. Researches to analyze the networks focusing on the temporal changes of multivariate using limited penetrable horizontal visibility graph and multiscale analysis have recently been conducted [79–83]. Investing the dynamic fluctuation of the subnet structure from the viewpoint of network with reference to such a method is also an interesting point. We expect that understanding this type of relationship would be a step toward a richer understanding of a mechanism of chaotic itinerancy within neural and behavioral dynamics.

In the current model, we have not yet introduced any learning methods to adapt a new environment or task because the purpose of our research is to understand the role and potential of the coupled dynamics of the body and the brain itself for spontaneous behaviors to emerge. However, the brain of humans and animals as self-organizing systems, might be reconstructed via their experiences and information from the outside. The changes influence their behavior and induce different experiences. How wired and information networks change through the interplay between behavior and a self-organized system in an environment and how it influences behaviors are interesting topics in developmental science. Several studies showed that changes in wired [84] and information networks [85, 86] in the brain occur according to age. These changes may play an important role in the emergence of behavior and functions of a human [87]. Another important future issue is understanding how various spontaneously emergent behaviors through the current model can be used to adapt to the environment or help motor development. Several studies showed that the diversity of behavior is effective for a fast learning of a new behavior rather than noise-like behaviors [88–90]. These studies also discussed the relationship between the diversity of the behavior in early infants and subsequent motor development [10]. We expect that our approach with learning methods, such as reinforcement learning for goal-directed behaviors, or Hebbian learning to learn corresponding behavior to the environment, will not only provide new insights into development and higher cognitive functions, but also adapt to engineering applications, such as the generation of robot behavior.

We used a simplified body and environment as we attempt to reveal the role of the topology of wired and information networks for the emergence of diverse movement patterns. However, in a developmental process, both the brain and the body changes, and a different behavior is observed according to age (e.g., crawling, standing, or grabbing an object using one or two hands). Several studies showed the importance of both the physical body and the environment for the emergence of movement patterns. For example, in an experimental study, Thelen and Smith [91] demonstrated the reappearance of a stepping movement in an infant following the changes in the body and the environment. In a simulation-based study regarding the morphological aspects of the body, Mori and Kuniyoshi [34] showed that a human-like distribution of tactile sensors induces human-like behaviors. Therefore, we expect that these morphological changes will induce different movement patterns and lead to a deeper understanding of the relationship of the brain, body, and environment. Furthermore, we also expect that multiple sensors will induce the emergence of a subnetwork with a specific role, such as a motor area or a sensor area in the brain.

## Conclusion

The purpose of this study is to understand the role and dynamics of the body and brain dynamics in bodily chaotic itinerancy, which emerges from the interaction between the body and the brain from the viewpoint of the diversity of behavior and the network. We address this issue by conducting a simulation using a musculoskeletal model and a nonlinear oscillator network to represent the body and brain dynamics, respectively. We then analyze the emerged behavior with dynamics using he information and complex network theories.

Through the experiment using different topological wired networks and sensor ratios, we showed that the number of emerged movement patterns is increased and restricted by the sensor ratio, and its degree of increase is changed by the structural property of the wired network, especially the degree of nodes. Moreover, the result of the analysis of the information network showed that the clustering coefficient and the shortest path length length in the information network have a negative and positive relation with the duration of the movement pattern, respectively, regardless of the structure of the wired network. We also suggested that the global interactions between the subnetworks with the active interaction and the body induce a transition between the stable movement patterns in chaotic itinerancy of bodily behaviors.

The current study showed the importance of the structural property of the brain and coupled dynamics from the interaction between the body and the brain for the emergence of diverse behaviors. We showed a possible mechanism of bodily chaotic itinerancy by revealing different structures and interactions of the information network behind the emergence of behaviors and their transitions. Future studies should address how wired and information networks change and affect human behavior in a developmental process using the entire musculoskeletal system of an infant. We believe such studies will offer new insights into understanding human behavioral development.

## Supporting information

S1 Video Example of the transition of a state in the dimensionality-reduced space by laplacian eigenmaps (WS model (p = 0.05) with randomly distributed weights, tonic input = 0.4, and sensor ratio = 0.3). The blue dots represent unstable movement patterns. The dots with other colors represent different stable movement patterns. The black empty circles indicate the current state of the movement pattern at that time.
(MOV)

S1 Fig Number of movement patterns for uniform weights. The tonic input for each graph is 0.4 (A), 0.45 (B), 0.5 (C), or 0.55 (D). The x-axis indicates the sensor ratio necessary *α* in Eq (3) to control the proportional influences between the body and the network. *** *p* < 0.001, ** *p* < 0.01, * *p* < 0.05 and ⋅ *p* < 0.1 indicate a statistically significant differences between the wired networks through the ANOVA test.
(EPS)

S2 Fig Number of movement patterns for randomly distributed weights. The tonic input for each graph is 0.4 (A), 0.45 (B), 0.5 (C), or 0.55 (D). The x-axis indicates the sensor ratio necessary *α* in Eq (3) to control the proportional influences between the body and the network. *** *p* < 0.001, ** *p* < 0.01, * *p* < 0.05 and ⋅ *p* < 0.1 indicate a statistically significant differences between the wired networks through the ANOVA test.
(EPS)

S3 Fig Number of stable movement patterns for uniform weights. The tonic input for each graph is 0.4 (A), 0.45 (B), 0.5 (C), or 0.55 (D). The x-axis indicates the sensor ratio necessary *α* in Eq (3) to control the proportional influences between the body and the network. *** *p* < 0.001, ** *p* < 0.01, * *p* < 0.05 and ⋅ *p* < 0.1 indicate a statistically significant differences between the wired networks through the ANOVA test.
(EPS)

S4 Fig Number of stable movement patterns for randomly distributed weights. The tonic input for each graph is 0.4 (A), 0.45 (B), 0.5 (C), or 0.55 (D). The x-axis indicates the sensor ratio necessary *α* in Eq (3) to control the proportional influences between the body and the network. *** *p* < 0.001, ** *p* < 0.01, * *p* < 0.05 and ⋅ *p* < 0.1 indicate a statistically significant differences between the wired networks through the ANOVA test.
(EPS)

S5 Fig Maximum duration of the movement pattern for uniform weights. The tonic input for each graph is 0.4 (A), 0.45 (B), 0.5 (C), or 0.55 (D). The x-axis indicates the sensor ratio necessary *α* in Eq (3) to control the proportional influences between the body and the network. *** *p* < 0.001, ** *p* < 0.01, * *p* < 0.05 and ⋅ *p* < 0.1 indicate a statistically significant differences between the wired networks through the ANOVA test.
(EPS)

S6 Fig Maximum duration of the movement patterns for randomly distributed weights. The tonic input for each graph is 0.4 (A), 0.45 (B), 0.5 (C), or 0.55 (D). The x-axis indicates the sensor ratio necessary *α* in Eq (3) to control the proportional influences between the body and the network. *** *p* < 0.001, ** *p* < 0.01, * *p* < 0.05 and ⋅ *p* < 0.1 indicate a statistically significant differences between the wired networks through the ANOVA test.
(EPS)

S1 File Values used in Figs 4 and 5. (XLSX)

S2 File Values used in Fig 6. (XLSX)

S3 File Average and standard deviation in Figs 7 and 8. (XLSX)

S4 File Values used in Fig 9. (XLSX)

S5 File Values used in Figs 10 and 11. (XLSX)
